# Species-conserved reconfigurations of brain network topology induced by ketamine

**DOI:** 10.1038/tp.2016.53

**Published:** 2016-04-19

**Authors:** R Becker, U Braun, A J Schwarz, N Gass, J I Schweiger, W Weber-Fahr, E Schenker, M Spedding, C Clemm von Hohenberg, C Risterucci, Z Zang, O Grimm, H Tost, A Sartorius, A Meyer-Lindenberg

**Affiliations:** 1 Research Group Translational Imaging, Department of Neuroimaging, Central Institute of Mental Health, Medical Faculty Mannheim, Heidelberg University, Mannheim, Germany; 2 Department of Psychiatry and Psychotherapy, Central Institute of Mental Health, Medical Faculty Mannheim, Heidelberg University, Mannheim, Germany; 3 Tailored Therapeutics Neuroscience, Eli Lilly and Company, Indianapolis, IN, USA; 4 Department of Psychological and Brain Sciences, Indiana University, Bloomington, IN, USA; 5 Department of Radiology and Imaging Sciences, Indiana University, Indianapolis, IN, USA; 6 Neuropsychiatry Pole of Therapeutic Innovation, Institut de Recherches Servier, Croissy-sur-Seine, France; 7 Spedding Research Solutions, Paris, France; 8 CNS Biomarker, Pharmaceuticals Division, F. Hoffmann-La Roche, Basel, Switzerland

## Abstract

Species-conserved (intermediate) phenotypes that can be quantified and compared across species offer important advantages for translational research and drug discovery. Here, we investigate the utility of network science methods to assess the pharmacological alterations of the large-scale architecture of brain networks in rats and humans. In a double-blind, placebo-controlled, cross-over study in humans and a placebo-controlled two-group study in rats, we demonstrate that the application of ketamine leads to a topological reconfiguration of large-scale brain networks towards less-integrated and more-segregated information processing in both the species. As these alterations are opposed to those commonly observed in patients suffering from depression, they might indicate systems-level correlates of the antidepressant effect of ketamine.

## Introduction

Ketamine, a potent *N*-methyl-d-aspartate (NMDA)-receptor antagonist, has spurred considerable interest in the preclinical as well as clinical applications. Its effects range from anesthesia after acute application of high doses to psychomimetic symptoms, derealization and cognitive disruption,^1, 2^ as well as relatively rapid but long-lasting antidepressant action after acute administration of lower doses.^3, 4, 5^ Furthermore, ketamine is widely studied in both human and laboratory animals as a translational pharmacological model of glutamatergic (dys-)function in schizophrenia.^1, 6, 7, 8^

Several studies have probed the underlying neurobiological substrates of these actions in humans^2, 9, 10, 11, 12^ and animals^6, 9, 13^ using neuroimaging approaches, but have largely focused on specific brain regions^12^ and their interactions. The first explicit demonstration of the translational potential of these methods showed altered prefrontal–hippocampal coupling after ketamine application in rats and humans, using a functional magnetic resonance imaging (fMRI) resting-state functional connectivity approach.^9^

However, given the ubiquity of NMDA receptors throughout the brain,^14^ it is likely that ketamine modulates large-scale neural networks on the system level, beyond alterations in single brain regions or specific circuits.

Developing translational biomarkers that are able to capture these global reconfigurations in a biologically meaningful way is a priority for drug research in psychiatry.^14, 15^ The application of network analysis in combination with task-independent brain imaging has been suggested as a key analytical tool, as network organization is closely linked to the brain function.^16, 17^ Providing a formal representation of brain function, network analysis allows these fundamental neurobiological organizational principles to be quantified across species. Recent studies have successfully established graph theoretical metrics as reliable^18, 19, 20, 21^ and sensitive biomarkers of normal^22, 23^ and psychopathology-associated network function,^22, 24^ both in humans and animals. Previous work also shows key network metrics to be conserved across species^25^ and comparable between biological neuronal networks and computers,^26^ further suggesting that this approach may be useful for translational research and biotechnological development.

Our aim was to investigate how acute ketamine challenge modulates the topological characteristics of the brain-wide functional connectome in both rats and humans, and to assess the consistency of the large-scale network changes across species.

## Materials and methods

### Subjects and ketamine application

Human fMRI data were acquired in 23 healthy individuals in a subject- and observer-blind, placebo-controlled, randomized three-period cross-over study, as previously reported.^9^ The subjects were invited in a fixed interval of 7 days with each scanning session taking place at approximately the same time of day. The actual measurements took place with a mean interval of 7.7 days (s.d.: 2.5; range: 4–16 days). On each of the three scanning visits, and approximately 60 min before the start of the MRI scan, the subjects received intravenous cannulation followed by an infusion scan via a certified intravenous pump (Braun Medical, Melsungen, Germany). The subjects received counterbalanced single intravenous doses of either saline (placebo condition), ketamine (0.5 mg kg^−1^ body weight) or scopolamine (4 μg kg^−1^ body weight) following previously published protocols.^27, 28^ All the study participants and personnel involved in the experiments were blind to the respective substance given. Individual doses of ketamine hydrochloride were adjusted to the body weight (0.5 mg kg^−1^) following previously published protocols,^29^ diluted in saline and applied over 40 min. The placebo condition consisted of a 40-min saline infusion. During the infusion, the subjects were seated comfortably in a chair under the supervision of a board-certified psychiatrist. To avoid order effects, the sequence of substance applications was randomly permutated across all the 23 participants. The MRI scanning took place after drug administration, with the resting-state measurement starting approximately 20 min after the end of the infusion. The data from the scopolamine challenge were not analyzed for the current report.

All the participants provided written informed consent for the study approved by the local ethics committee (Medical Faculty Mannheim, University of Heidelberg, Mannheim, Germany). A total of 23 participants completed the study (11 female, mean age: 25.13±2.51 years, mean body weight: 70.24±11.56 kg, mean height: 1.75±0.80 m, mean body mass index 22.76±2.71). One subject that initially participated was excluded from the study, as she was not able to complete all the three required MRI measurements.

As this was an exploratory study, no formal power or sample-size estimation was performed. The group size was selected on the basis of prior experience and literature reports of pharmacological-fMRI studies in healthy subjects, and is toward the high end of the range of sample sizes typically used.

### Animals and ketamine application

Eighteen Sprague Dawley male rats (weight: 373–447 g, age: 10–11 weeks; Janvier Laboratories, Le Genest-St-Isle, France) were used for the fMRI experiments. The animals were housed under controlled conditions (19–23 °C, 40–60% humidity) with a 12:12 h light–dark cycle (lights on at 0700 h).

All the procedures were performed according to the regulations covering animal experimentation within the European Union (European Communities Council Directive 86/609/EEC) and within the German Animal Welfare Act and were approved by the German animal welfare authorities (Regierungspräsidium Karlsruhe). The rat rs-fMRI data were originally analyzed using a seed-based approach, the results of which have been reported elsewhere.^6^ As that study was exploratory in nature, no formal power or sample-size estimation was performed, but the group sizes (*N=*9 per group) are toward the high end of the range typically used in rat fMRI experiments. The exploratory nature of the experiment was explicitly mentioned in the discussion section of ref. 6. No blinding was done, as for the given type of experiment it is not possible: ketamine solution had to be prepared before each experiment depending on the dose.

The experimental design comprised two groups of *N=*9 rats each. In one group, S-ketamine (Ketanest, Pfizer Pharma, Berlin, Germany) was injected subcutaneously at a dose of 25 mg kg^−1^ dissolved in saline (total volume 1 ml kg^−1^). The second group received the same volume of vehicle (saline). The rats were assigned to the groups randomly. The order of ketamine and saline injections was randomized across the animals and time of day. The fMRI resting-state measurements were acquired immediately before and starting at 15 min after the ketamine/vehicle injection.

**Figure 1:**
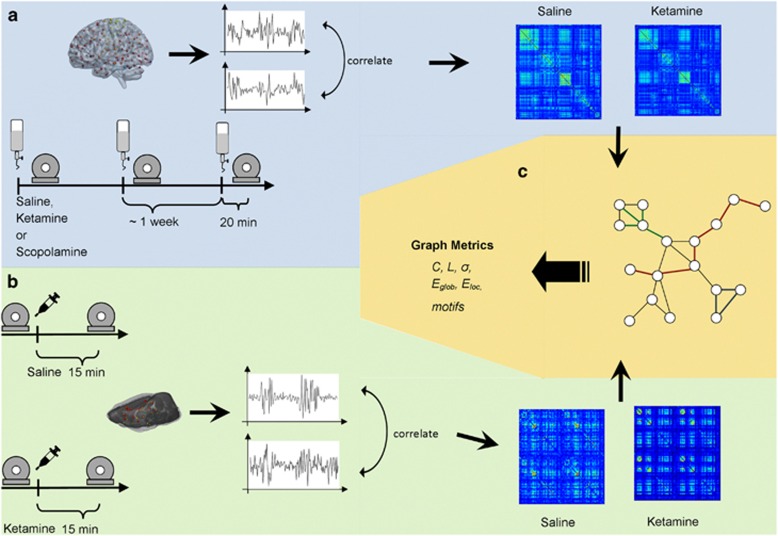
Study protocols and methods. (**a**) Human (light blue): the subjects received either saline, ketamine or scopolamine via infusion over 45 min, 20 min before each scanning session. BOLD fMRI resting-state data were acquired and the time series of 270 brain regions were extracted. Correlated, these yielded two correlation matrices for each subject. (**b**) Rats (light green): the two groups of animals received either ketamine or saline injection. BOLD resting-state data were acquired before and 15 min after the injection. The time series of 90 brain regions were extracted and correlated, resulting in one correlation matrix for each animal. (**c**) Both human and animals matrices were thresholded and binarized to extract the underlying topological network structure. Subsequently, graph metrics such as degree (green lines in the depicted network), path length (red lines) and clustering coefficient (blue) were computed. BOLD, blood-oxygen-level dependent; fMRI, functional magnetic resonance imaging.

These animals represent two groups from the study previously reported.^6^ The 25 mg kg^−1^ group was selected for the present analysis as it yielded plasma concentrations closest to those obtained in the human study.^9^ The rat data were re-analyzed using methods as closely aligned as possible to those used for the human data (see Figure 1).

### Data acquisition and preprocessing—human fMRI

Blood-oxygen-level-dependent fMRI was acquired at a 3-Tesla MR scanner (Siemens TIM-Trio, Erlangen, Germany), with the Syngo MR VB17 Software, maximum 45 mT m^−1^ (*z* axis) and 40 mT m^−1^ (*x* and *y* axis) gradient strength, a 32-channel head-coil and an echo-planar imaging sequence with full brain coverage with the following parameters: repetition time=1790 ms, echo time=28 ms, 34 oblique slices (aligned to the anterior commissure–posterior commisure plane) in descending acquisition order, 3 mm slice thickness, +1 mm gap, flip angle=76°, FoV=192 mm, 64 × 64 matrix, 3 × 3 mm in-plane voxel size and 332 volumes. Preprocessing of functional data consisted of slice time correction, realignment and smoothing by a 6 mm full-width half maximum Gaussian kernel (FSL 5.0.6) followed by noise correction with the AROMA framework.^30^ In short, this includes removal of noise that is related independent by means of an automated detection of noise components based on their time course correlation with movement parameters, as well as their high frequency fraction and the overlap of their maps with brain edges and cerebrospinal fluid maps (for details, see ref. 30). The data were afterwards corrected for nuisance covariates of white matter and cerebrospinal fluid signal as well as the six motion parameters obtained during the realignment.^30^ The data were finally normalized to MNI standard space echo-planar imaging template (SPM8). A total 264 whole-brain functional nodes were extracted from 5-mm spheres around coordinates defined by Power *et al.*^31^ As the Power atlas does not cover the hippocampus, amygdala and nucleus accumbens, we included additional six bilateral nodes of interest based on the meta-analytical data.^32, 33, 34^ In a final step, each node's time series was bandpass filtered at a frequency range of 0.01 to 0.15 Hz.

### Data acquisition—rat fMRI

The experiments were conducted at a 9.4-Tesla MRI scanner (94/20 Bruker BioSpec, Ettlingen, Germany) with Avance III hardware, BGA12S gradient system with the maximum strength of 705 mT m^−1^ and Paravision 5.1 software. Transmission and reception were achieved using a linear whole-body volume transmitter coil combined with an anatomically shaped four-channel receive-only coil array for the rat brain.

The rats were anesthetized under 4% isoflurane (Baxter Deutschland, Unterschleissheim, Germany) in a mixture of N_2_ (70%)/O_2_ (30%). After positioning in the scanner (head first, prone), 2.5% isoflurane was provided for adjustments. Then, a bolus of 0.5 ml medetomidine solution (Domitor, Janssen-Cilag, Neuss; 0.07 mg kg^−1^ subcutaneously) was administered; isoflurane was slowly discontinued within the next 10 min, after which a continuous infusion of medetomidine solution started at 0.14 mg kg^−1^ h^−1^ rate.

The breathing and cardiac signals were monitored using a respiration pad placed beneath the chest (Small Animal Instruments, Stony Brook, NY, USA) and a pulse oximeter attached to the hindpaw, respectively. A signal breakout module (Small Animal Instruments) and a four-channel recorder (Velleman, Gavere, Belgium) were used to record signals (10-ms resolution).

The MRI acquisition protocol for each animal comprised a Field Map and a rs-fMRI measurement. To acquire the rs-fMRI time series, an echo-planar imaging sequence was used with the following parameters: repetition time/echo time 1700/17.5 ms, flip angle 60°, one segment, 29 coronal slices (ascending slice order), 96 × 96 imaging matrix, field of view 35 × 35 mm^2^, slice thickness 0.5 mm with 0.2 mm interslice gap, in-plane voxel dimension 0.365 mm, 300 acquisitions over 8.5 min. The slice stack covered the brain from the cerebellum to the posterior olfactory bulb.

The preprocessing of the data included correction for field inhomogeneities (SPM8), regression of movement parameters (FSL, version 4.1), filtering of respiratory and cardiac signals (Aztec^35^), slice timing correction (SPM8) and band pass filtering (0.01 to 0.1 Hz, using Analysis of Functional NeuroImages (AFNI) software). In addition, the images were spatially normalized (SPM8) to a rat brain template with co-registered atlas in the Paxinos stereotactic coordinate system^36^ and finally the signal from the cerebrospinal fluid was filtered out. A single time series was extracted for each of the 90 anatomically defined regions of interest (Schwarz *et al.*^36^) by averaging single-voxel time series over all the voxels in a region of interest.

### Network analysis—humans and rats

The brain networks for humans and rats were constructed by computing the Pearson correlation coefficient between the time series extracted from each pair of regions of interest. The correlation coefficients were transformed to Fisher's *z*-scores. Subsequently, networks were created by binarizing these correlation matrices. As there is no current consensus on the selection of network density (fraction of all possible links that is actually present), we computed all graph metrics over a range of densities (0.05 to 0.2 in steps of 0.01—for details, see Supplementary Material) as previously described.^13, 19, 21, 31^ The selection of density thresholds was led by two methodological considerations. On one hand, we wanted our analysis to be sensitive to subtle alterations in network topology. Because of the large number of possible connections and the relatively small number of connections actually being different under ketamine, a threshold beyond 0.2 would significantly impair this sensitivity. One the other hand, given the high degree of network fragmentation (that is, the number of disconnected nodes) at lower densities, we chose a density of 0.05 as the lowest cutoff to ensure a reasonable degree of connectedness.

This procedure normalizes all networks to have the same number of links and removes the effect of global differences in correlation strength. Standard network parameter computation was implemented in MATLAB, using the Brain Connectivity Toolbox.^37^ For each subject and each density level, we computed the following network parameters: clustering coefficient *C*, characteristic path length *L*, small-worldness *σ*, global efficiency *E*_glob_ and local efficiency *E*_loc_. The details on the computation and interpretation of the specific graph metrics can be found in the Supplementary Material.

Furthermore, we calculated the networks' motif frequencies, also using functions implemented in Brain Connectivity Toolbox, for a sequence of eight motifs consisting of (undirected) connections of three to four nodes.

### Network Based Statistics—humans and rats

Although network analysis can detect changes in the topological organization of brain networks, we also aimed to test for global differences in absolute connectivity. To that end, we used the Network Based Statistics (NBS) toolbox^37, 38^ to identify alterations in specific connections after ketamine application. The NBS deals with the multiple comparison correction for 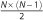 tests by evaluating the null hypothesis on the level of connected subcluster rather than individually for each connection. Using a corrected *P*-level of 0.05, we report the largest connected subclusters identified by NBS.

### Statistical analysis and hypothesis testing

Instead of testing each metric at each density level, we calculated the area under the curve for each metric, motif frequency and subject. This yields density summary estimators for each network metric and avoids the still-unresolved question of multiple comparison correction in the modern network analysis.^39^ Subsequently, we tested the areas under the curve of each metric using paired and independent sample *t*-tests. For comparisons in humans, a paired *t*-test was conducted, whereas independent two-sample *t*-tests were used for the rats as they consisted of two different groups. All reported *P*-values were <0.05, false discovery rate (FDR)-corrected for multiple comparisons unless otherwise specified.

## Results

### Alterations of network metrics by ketamine in humans

**Figure 2:**
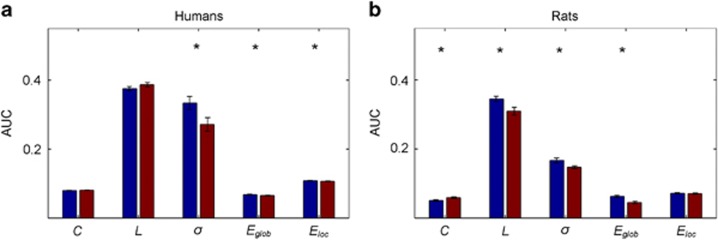
Topological reconfigurations after ketamine application. Means of area under curve (AUC) for each calculated graph metrics for humans (**a**) and rats (**b**) after saline application (blue) and ketamine (red). Asterisks denote statistically significant differences (*P<*0.05, FDR corrected) between saline and ketamine. *C*, clustering coefficient; *E*_glob_, global efficiency; *E*_loc_, local efficiency; FDR, false discovery rate; *L*, path length; *σ*, small-worldness coefficient.

After the application of ketamine, human brain networks showed a decrease in the global and local efficiency compared with placebo (*P*_FDR_<0.05), as well as a decrease in the small-world coefficient (*P*_FDR_<0.05). An increase in the characteristic path length was found only as trend (*P*=0.08). Further, ketamine induced a significant increase in cyclic motifs (*P*_FDR_<0.05), whereas the acyclic motifs were not significantly altered (see Figure 2).

### Alterations of network metrics by ketamine in rats

**Figure 3:**
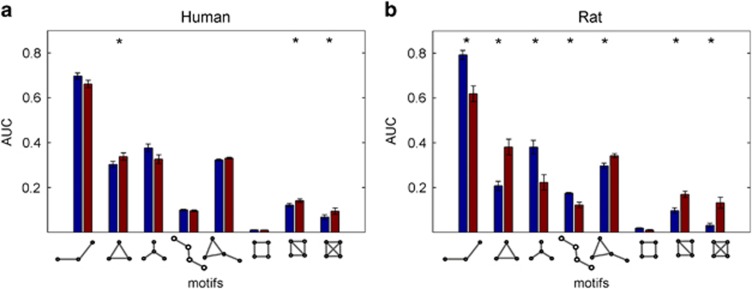
Area under curves of network motif frequencies for undirected three- and four-node motifs. For both humans (**a**) and rats (**b**), red bars indicate mean values for the ketamine groups, and blue bars indicate mean values for the controls; asterisks denote statistically significant differences between the groups (*P<*0.05, FDR corrected). AUC, area under the curve; FDR, false discovery rate.

After the application of ketamine, the brain networks of rats showed an increase in clustering coefficient compared with placebo (*P*_FDR_<0.05), as well as an decrease in the average path length (*P*_FDR_<0.05). This is reflected in the decrease in global efficiency (*P*_FDR_<0.05). Sigma (*σ*), the small-world coefficient also decreased (*P*_FDR_ <0.05). As in humans, networks after ketamine showed an increased abundance of cyclic motifs (*P*_FDR_<0.05) and additionally a significant decrease in acyclic motifs (see Figure 3).

To provide further evidence that the observed group differences between saline and ketamine conditions were indeed induced by the drug challenge (and are not driven by accidental differences between both the groups), we repeated our analysis in both the groups before applications of either ketamine or saline. As expected, we could not detect any difference between the groups in any graph metric (all *P*>0.5, see Supplementary Figure 1).

### Alterations of functional connectivity by ketamine in humans

**Figure 4:**
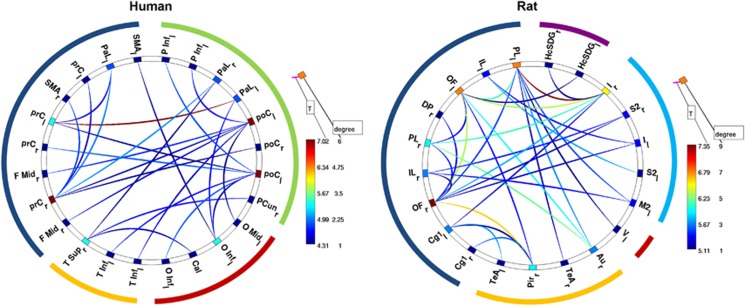
Alterations of functional connectivity after ketamine application. Ketamine-related increase in connectivity in humans (left) and rats (right). The line color represents the statistical significance as indicated by the respective *T*-statistics. The color of nodes indicates the number of altered connections for the respective node. The outer colored circle represents the brain region's assignment: blue=frontal cortex, yellow=temporal, red=orbital cortex, green=parietal cortex, magenta=hippocampal formation. The areas colored in lighter blue stretch along an anterior-posterior axis compassing multiple brain regions. Human: Cal, calcarine sulcus; F Mid, frontal middle cortex; O Inf, occipital cortex inferior sulcus; O Mid, occipital cortex middle sulcus; PaL, paracentral lobule; PCun, precunes; poC, postcentral sulcus; prC, precentral sulcus; SMA, supplemental motor area; T inf, temporal cortex inferior sulcus; T sup, temporal cortex superior sulcus. Rat: Au, auditory cortex; Cg1, cingulate cortex; HcSDG, hippocampus subiculum/dentate gyrus; I, insular cortex; IL, infralimbic cortex; M2, secondary motor cortex; OF, orbitofrontal cortex; Pir, piriform cortex; PL, prelimbic cortex; S2, secondary somatosensory cortex; TeA, temporal association area; V, visual cortex. Subscript indicates left (l) or right (r) hemisphere.

The NBS revealed a subnetwork consisting of 24 nodes and 24 increased connections with a *T*-statistic of *T*>4.4 induced by infusion of ketamine (*P*-corrected <0.05). This subnetwork predominantly comprised regions of the frontal cortex (~38%), parietal cortex (~33%), as well as occipital (17%) and temporal cortex (~13%). To further illustrate the importance of specific brain areas to the large-scale reconfiguration observed before, we evaluated the number of altered connections for each node (see Figure 4). Two frontal areas, namely frontal superior medial cortex and the middle frontal cortex, as well as the mid cingulate cortex exhibited the highest number of altered connections, identifying them as being prominently involved in ketamine-induced reconfigurations of brain networks.

### Alterations of functional connectivity by ketamine in rats

A subnetwork of 31 edges with a *T*-statistic of *T*>5.1 and 21 nodes was identified in rats after ketamine application (*P*-corrected <0.05). Increased connections could be mainly detected in the infralimbic, prelimbic and orbital regions of the frontal cortex, as well as the insular cortex (see Figure 4). Several cingulate and somatosensory areas also showed increased connectivity. The largest changes in the number of connections occurred in the frontal and insular cortices. As in humans, the frontal cortex was most strongly affected by ketamine-induced network reconfiguration.

## Discussion

The human and rat brains, despite all their differences, have shown some remarkable similarities in the spatial, functional and topological characteristics of intrinsic network architecture.^18, 25, 40^ The network analysis has been proposed as a promising tool for translational neuroscience and especially cross-species drug discovery,^15^ but studies utilizing the translational power of these approaches^15, 41^ are rare.

Here, we used network analysis to study the global topological reconfiguration of human and rat brain networks in response to a pharmacological challenge with subanesthetic doses of ketamine.

### Consistent reconfiguration of brain networks in humans and rats

After ketamine infusion, brain networks in both humans and rats showed an increase in overall connectivity, consistent with the results from previous studies.^1, 42, 43^ In both species, the frontal cortex showed particularly prominent connectivity changes, which may be related to our previous findings of altered prefrontal–hippocampal connectivity.^9^ Regarding the other affected brain regions, between-species concordance was incomplete. However, it should be noted that NBS is suitable for identifying cluster-wide connectivity changes, not for making inferences on specific connectivity pairs. This precludes detailed interpretation of the individual regions displaying altered connectivity in our data.

The connectivity increase was accompanied by a topological reconfiguration of large-scale brain networks towards a conformation characteristic of less-integrated and more-segregated information processing, as evidenced by decreases in global efficiency and a decrease in small-worldness. A higher abundance of cyclic motifs points towards a network organization more tuned to local processing and less suitable for efficient long-range communication in both the species. This is further supported by the decreased occurrence of acyclic motifs in rats, although this finding was not significant in humans. In line with a previous study using a different imaging modality, cross-animal correlations rather than within-subject temporal correlations and a different NMDA receptor antagonist,^44^ we observed a loss of small-worldness properties after ketamine injection. Our study therefore adds further support to the idea of a compromised functional integration in NMDA receptor dysfunction models of disease. It is interesting to note that a previous study from our group using the same human and rat data as in the current work reported an increased prefrontal–hippocampal coupling after NMDA receptor blockade to be a cross-species phenotype altered by ketamine.^9^

Although a direct statistical comparison between the species would be misguided for several reasons, the placebo-controlled design of both studies enabled us to compare findings between species qualitatively. Overall, we found highly concordant and directionally consistent network reconfiguration in humans and rats. In both the species, we observed an increased total connectivity. Topologically, we detected a less globally integrated and more-segregated network architecture following ketamine administration.

### Discrepant findings in humans and rats

Interestingly, although motif analysis revealed a significant increase of cyclic motifs after ketamine infusion in both the species, we found no difference in the clustering coefficient in humans. This might be a consequence of triangles being introduced primarily around the nodes with a high degree, for which the clustering coefficient is less sensitive due to its normalization by the number of all possible triangles.

Surprisingly, in rats, we observed a decrease in the path length after ketamine application, contrasting with effects on other parameters indicating a less-integrated network architecture. However, this might be a result of our focus on sparsely connected networks, which often suffer from a higher fragmentation to which the characteristic path length (in contrast to global efficiency) is very prone.

### Relation to mental disorders

Interestingly, the network reconfigurations identified here are opposite to network alterations in depressed patients, who show increased global efficiency and small-worldness compared with healthy controls.^45, 46^ We hypothesize that this may indicate a network mechanism supporting the action of ketamine as rapidly acting antidepressant. This idea is further supported by a previous study showing ketamine effects on network topology opposite to those observed in depressed subjects.^13^

The studies investigating brain network properties in schizophrenia consistently report reduced total connectivity, as well as higher integration (for example, increased local efficiency, decreased characteristic path length) and less local processing (for example, decreasing clustering coefficient and local efficiency).^47, 48^ These findings are in contrast to the observed network reconfigurations in our data, suggesting that acute ketamine application rather resembles a different glutamatergic state than observed in chronic schizophrenia.

### Limitations

Despite our efforts to align protocols of the human and rat studies, there still remain some differences. First, ketamine was applied differently—subcutaneous bolus injection in rats versus intravenous continuous infusion in humans—and therefore might evoke different biological responses. Further studies are urgently needed, as application protocols might explain clinically diverse effects and could account for the heterogeneous results in previous human^11, 42^ and rat^6^ studies. Second, in rats, anesthesia as well as interactions of the anesthetic agent with ketamine might also influence the behavior of brain networks, although previous studies have shown a high correspondence of human and rat functional brain networks.^9, 49^ A further potential limitation results from the different number of nodes used in humans and rats. However, by choosing a proportional thresholding approach, we aimed at examining the changes in network architecture independent of the absolute number of links in a given network. Because of the thresholding approach, our results are specific to the chosen range of network densities (5–20%), which surely is not the only possible choice of densities. Along with network size, the composition of nodes is a possible restriction to comparability. In humans, 90% of the nodes are cortical, whereas in rats, the fraction is considerably smaller (40%), which reflects the substantially developed and differentiated cortex in humans. Despite this asymmetry in the fraction of cortical nodes, the results in both the species can be compared, thanks to the placebo-controlled design of the study in both species. We compare the changes in network organization qualitatively rather than absolute quantities of network metrics in both the species. Moreover, a closer look at the brain regions that experience most significant change in connectivity and number of connections reveals that in rats as well as humans, the effects of ketamine occur (almost) exclusively in the cortical regions (see Figure 4). Another potential confound is the influence of the cerebral blood flow (CBF) increase induced by ketamine.^50^ The blood-oxygen-level-dependent signal correlation changes that we observed could include contribution from CBF increase *per se* or the differences in correlation may be modulated by CBF increase. Regrettably, we did not measure CBF independently and therefore cannot access its impact on network results. Given the promising results on ketamine effects on functional connectivity in this study (and others in recent literature), further studies examining the effect of CBF are recommended. Finally, while the animal study had a two-group design, the human study was designed as a placebo-controlled double-blind cross-over study. The missing control for between-subject variance in the animal study might account for the observed higher differences in rats, but a comparison between the groups before ketamine applications revealed no differences between network parameters (see Supplementary Material).

### Relevance for translational research and drug discovery

These findings are encouraging for translational research and its application to drug discovery. The use of global brain parameters reflecting functional network topology has the advantage of reducing complex, high-dimensional imaging data to a few summary measures, reducing the multiple comparisons burden and avoiding the need to pre-specify specific brain regions or connections. The latter is valuable, and the involvement of specific brain systems is important to increase biological understanding, but can be difficult to apply with confidence, especially when dealing with novel compounds or mechanisms. The global brain network parameters may thus represent a more robust end point for translational functional imaging at the early phase of drug discovery and development. Importantly, the similarities in the pharmacological effects across species suggest that preclinical experiments can be used to inform early clinical phase biomarker studies. That said, the differences in the observed effects serve as a reminder that effects in humans are unlikely to be a perfect simulacrum of those observed in the preclinical setting. This observation is consistent with other recent translational pharmacological imaging studies, where consistency between rodent and human effects was high but not complete.^51^

Another important caveat is that ketamine is a highly psychoactive compound. Novel compounds being developed as potential medicines are unlikely to induce such strong subjective effects *per se*, and their effects on brain functional connectivity may be more subtle. The sensitivity of network topology measures to a broader range of compounds and mechanisms remains to be established. However, as a pharmacological model of a hyperglutamatergic state, ketamine-induced changes in functional connectivity may serve as an example of brain function perturbation that can be reversed by pre-treatment with compounds of interest.^10, 52^ Finally, the sensitivity of results to methodological aspects such as brain parcellation scheme, image post-processing variations, choice of connectivity metric and thresholding or weighting scheme remains an important area of investigation.

## Conclusions

Using network analysis and task-independent brain imaging, we observed highly concordant cross-species topological reconfigurations of large-scale brain networks induced by acute ketamine administration in rats and humans. These changes indicated a pharmacologically induced shift towards a more-segregated, less-integrated network conformation and are opposite in valence to network topology changes observed in depressed subjects.

In conclusion, our results demonstrate the potential of these novel techniques for the identification of translational biomarkers for drug discovery.
