# Supplementary material for: Species-conserved reconfigurations of brain network topology induced by ketamine
Source: Transl Psychiatry. 2016 Apr 19;6(4):e786–. doi: 10.1038/tp.2016.53 (PMC4872411; doi:10.1038/tp.2016.53)
Supplement: Supplementary Information [file tp201653x3.doc]

Figure S1: Comparison of graph metrics in both rat groups before application of saline or ketamine

No difference between the saline (blue) and ketamine (red) group could be detected for any of the tested graph metrics. All p > 0.5 before corrections for multiple comparisons.

Figure S2: Comparison of graph metrics in humans after saline and ketamine for different frequency bands

Area under curves for each calculated graph metrics for humans with a bandpass filter of 0.01 to 0.15 Hz (A) and 0.01 to 0.15 Hz (B) after saline application (blue) and ketamine (red). Asterisks denote statistically significant differences (p<0.05, FDR corrected)  between saline and ketamine. C = clustering coefficient, L = path length, σ = small-worldness coefficient, Eglob = global efficiency, Eloc = local efficiency.
